# Late recurrences of germ cell malignancies: a population-based experience over three decades

**DOI:** 10.1038/sj.bjc.6603014

**Published:** 2006-02-28

**Authors:** J Oldenburg, G C Alfsen, H Wæhre, S D Fosså

**Affiliations:** 1 Faculty of Medicine, University of Oslo, Oslo, Norway; 2 Department of Clinical Cancer Research, Rikshospitalet-Radiumhospitalet Trust, Oslo, Norway; 3 Department of Pathology, Rikshospitalet-Radiumhospitalet Trust, Oslo, Norway; 4 Department of Surgical Oncology, Rikshospitalet-Radiumhospitalet Trust, Oslo, Norway

**Keywords:** late relapse, late recurrence, testicular cancer, extragonadal germ cell cancer, EGGCT, seminoma, non-seminoma, retroperitoneal lymph node dissection

## Abstract

The purpose of this study was to explore the incidence of late relapse in patients with malignant germ cell tumour (MGCT) in a population-based series, with emphasis on the mode of detection, survival, and the relevance of histological findings. The clinical records from a population-based cohort of patients with seminoma (*n*=1123) or non-seminoma (*n*=826) were evaluated for late relapses. Twenty-five patients developed a late relapse. The cumulative 10-year incidence rate was 1.3%. All 10 seminoma patients, but only eight of 15 non-seminoma patients relapsed with vital malignant tumour (*P*=0.02). Teratoma or necrosis was found in seven of nine primarily chemotherapy-treated non-seminoma patients with normal tumour markers at late relapse. Six of nine patients operated with limited retroperitoneal lymph node dissection as part of the primary treatment had relapsed retroperitoneally outside the original operation field. The 10-year cause-specific survival was 68% in all patients, 50% in patients relapsing with vital malignant tumour and 100% in those with teratoma/ necrosis before or after salvage chemotherapy. The 10-year incidence rate of late relapses of 1.3% might reflect the true incidence rate in a population-based cohort of MGCT patients, with cure in at least half of them.

Late relapses of malignant germ cell tumours (MGCTs) are rare events. Most investigators define late relapses as recurrence at least 2 years after completion of successful primary treatment and differentiate between those occurring before and after 5 years (Baniel *et al*, 1995; Gerl *et al*, 1997; Shahidi *et al*, 2002; George *et al*, 2003; Dieckmann *et al*, 2005). Due to the rarity of late relapses, systematic reports on this topic are restricted to large referral-centres, and may therefore be comprised of late relapsing patients who have received their primary treatment elsewhere in addition to those who have had their primary treatment at the respective institution. Reported cure rates in patients with these often highly aggressive and chemotherapy-resistant tumours vary from 26 to 69% (Borge *et al*, 1988; Baniel *et al*, 1995; Gerl *et al*, 1997; Shahidi *et al*, 2002; George *et al*, 2003; Lipphardt and Albers, 2004; Dieckmann *et al*, 2005). In 1988, Borge *et al* (1988) from our hospital reported a crude late relapse rate of 1.5%, which is lower than that observed by most other investigators (Baniel *et al*, 1995; Gerl *et al*, 1997; Shahidi *et al*, 2002; George *et al*, 2003).

Some authors recommend regular follow-up of *all* patients with MGCT beyond the usual 5-year period, arguing that asymptomatic patients with early-detected relapses have a better prognosis compared to patients with symptoms (Gerl *et al*, 1997; George *et al*, 2003). However, regular follow-up of *all* patients with MGCT at specialist centres beyond 5 years might represent an unnecessary routine in the majority of men.

The purpose of the present study is to explore the true incidence rate, mode of detection, and outcome of late relapsing MGCT patients in a large population-based cohort of patients who had their primary treatment at the Norwegian Radium Hospital (NRH). We aim to examine the impact of the histology both at initial diagnosis and at late relapse on clinical course and outcome.

## PATIENTS AND METHODS

### Patients

Since 1970 post-orchiectomy treatment was performed at the NRH in all patients with a germ cell malignancy in the southern part of Norway. Roughly, half of Norway's MGCT diagnoses are made in the population from this geographic area. At referral, sections from the orchiectomy specimen are routinely reviewed. In order to assess the risk of late relapses after modern treatment of MGCT, all seminoma-patients referred to the hospital from January 1971 to December 1997, and all non-seminoma-patients referred from January 1980 (after the advent of cisplatin) to December 1997, were screened for late relapse, based on the hospital's patient registry. The registry separates patients with testicular cancer (TC) from those with extragonadal germ cell tumour (EGGCT) from 1980 onwards. Patients with TC or EGGCT were eligible for the present analysis if they: (1) had been considered tumour-free after their primary treatment at the NRH; and (2) had remained relapse-free for at least 30 months after the initial diagnosis (assuming a maximal treatment period of 6 months), but had developed a relapse thereafter. The event of a contra-lateral TC was not counted as a late relapse.

### Staging

Extragonadal germ cell tumours were not staged, but described as mediastinal or retroperitoneal primaries. For the purpose of the present study, patients with metastases were staged retrospectively according to the International Germ Cell Consensus Classification Group (IGCCCG) (1997) and the UICC-staging guidelines (Sobin and Wittekind, 2002).

### Treatment

The treatment strategies have been described previously (Fossa and Ous, 1985; Fossa *et al*, 2003) and are summarised in Table 1. Treatment for patients with *seminoma* had remained principally unchanged from 1971 to 1997. Radiotherapy was given in stage I and small volume stage II disease and chemotherapy was given for advanced seminoma, followed by post-chemotherapy radiotherapy up until 1987. Before 1980, chemotherapy consisted of alkylating agents. After 1980, cisplatin-based chemotherapy was used.

The advent of cisplatin-based chemotherapy at the end of the 1970s represented a major change in the treatment of *non-seminoma* patients. Up to 1990, unilateral template RPLND (retroperitoneal lymph node dissection) was used in stage I or small volume stage II disease (Fossa and Ous, 1985). From 1990 a surveillance policy for stage I was established. In metastatic patients, post-chemotherapy RPLND was initially performed bilaterally (Whitmore Jr, 1979). From 1986, ipsilateral unilateral template RPLND only was introduced, refined by nerve-sparing techniques, whenever possible, after 1990 (Donohue *et al*, 1990; Jacobsen *et al*, 1999).

### Follow-up

Patients, rendered tumour-free by primary treatment, were with increasing intervals followed for 5 years at the hospital's out-patient department with clinical-, radiological-, and biochemical examinations. Computed tomography (CT)-examinations were introduced in the 1980s and were performed predominantly during the first 2 years in patients following a surveillance policy or after treatment for advanced stages. Generally, no further follow-up was carried out beyond 5 years at the oncology centre, except in patients who were *ad hoc* considered risk patients. All patients with relapses were referred to our hospital for treatment.

### Clinical course

Date of initial diagnosis and of relapse, IGCCCG-stage (1997), UICC-stage (Sobin and Wittekind, 2002), tumour localisation at the time of relapse, date of start and type of primary and relapse treatment, and serum tumour markers before initial treatment and at time of relapse were retrieved from medical records. The mode of relapse detection was also noted (coincidental finding at routine follow-up or due to clinical symptoms). From 1985 onwards, a histological confirmation of the late relapse prior to salvage treatment was attempted. All available histological specimens were reviewed by one pathologist (GCA) and described according to the WHO classification system (Ulbright Thomas *et al*, 1999). At relapse the diagnosis of viable malignant tumour, that is, non-teratomous- and non-necrotic tumours, was based on histology or on AFP/HCG elevation. Patients with late relapses were treated on an individual basis at the responsible physician's discretion. Chemotherapy for relapses consisted principally of cisplatin-based combinations, preferentially using drugs that had not been employed previously in the respective patient.

### Statistics

The SPSS program version 12.0.2 was used for descriptive (median, range) and analytical methods (*t*-test, Mann–Whitney, *χ*^2^, Fisher's Exact, and Kaplan–Meier procedure and log-rank test for survival analysis). All patients were followed to date of death or, for surviving patients, to the cutoff date of the study (October 1st 2004). The incidence-observation time ranged from date of initial diagnosis of MGCT to date of late relapse diagnosis, patient's death or cutoff date, whichever came first. Survival-observation time ranged from date of late relapse diagnosis to patient's death or cutoff date. A *P*-value <0.05 was considered statistically significant.

## RESULTS

### Patients and incidence of late relapses

One thousand one hundred and twenty-three patients with seminoma and 826 patients with non-seminoma had their primary treatment at the NRH from 1971 and 1980, respectively, until December 1997. Twenty-five (1.3%, seminoma: 10, non-seminoma: 15) of these 1949 patients developed a late relapse (TC: 21, EGGCT: 4, Table 2). Their pre-relapse treatment consisted of platinum-based chemotherapy (seminoma: 3; non-seminoma: 14) or radiotherapy only (seminoma: 7). One patient following the surveillance policy.

**Figure 1:**
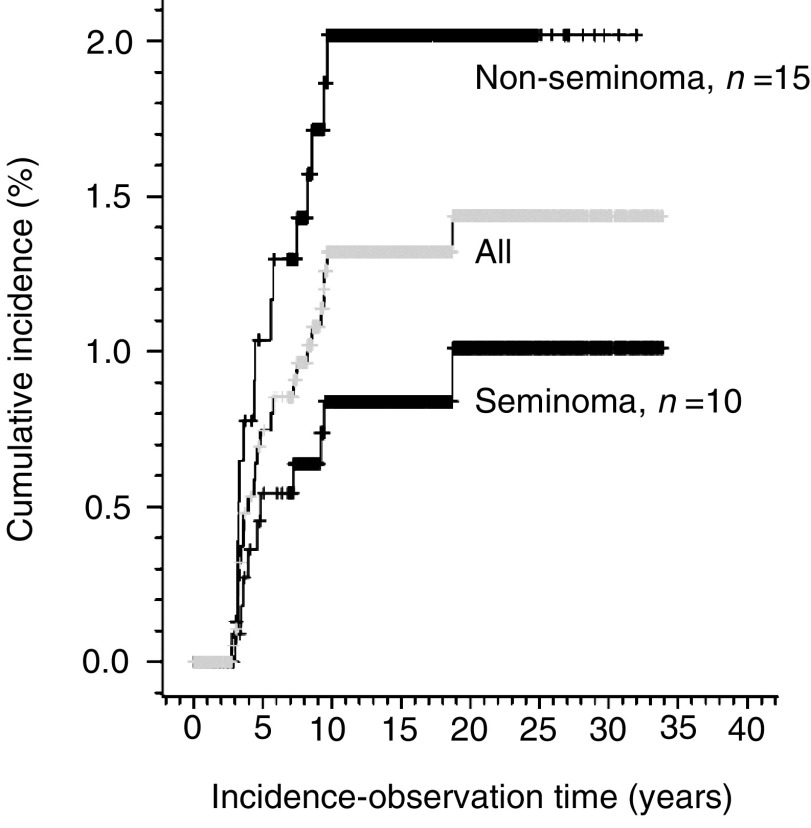
Cumulative late relapse incidence in 1949 MGCT patients.

The cumulative 10-year incidence rate of all 25 late relapses was 1.3% (95% CI: 0.8–1.8, after 20 years 1.4%, 95% CI: 0.9–2.0, Figure 1). All but two relapses were diagnosed within the first decade after the initial diagnosis of MGCT (Figure 1). The 10-year cumulative incidence rate for seminoma patients was 0.8% (95% CI: 0.2–1.3) and 1.9% (95% CI: 0.9–2.8) for non-seminoma patients (log-rank test, *P*=0.047). The 10-year incidence-rate of patients diagnosed in 1980 or later was 1.2% (95% CI: 0.6–1.7) for 1570 TC patients, and 6.0% (95% CI: 0.0–12.5) for 60 patients with EGGCT (log-rank test *P*<0.001). In patients who received primary chemotherapy (*n*=480), the cumulative 10-year incidence rate was 3.1% (95% CI: 1.4–4.8%). This was significantly higher than the comparable incidence rate of 0.7% in the remaining patients (*n*=1469) who did not receive primary chemotherapy (95% CI: 0.3–1.1%, log-rank test, *P*<0.001). Fifteen patients developed a recurrence more than 66 months after orchiectomy, that is, very late relapses. Their clinical parameters or survival did not differ from the others.

### Mode of detection and presentation of late relapses

Eleven patients were diagnosed symptom-free during scheduled routine follow-up examinations in 10 or during an assessment of long-term TC-survivors in one (#8, Table 3) (Fossa *et al*, 2003). Symptomatic patients presented later and with bigger masses compared to the former group (*n*=14, Mann–Whitney, *P*=0.013 and 0.025, respectively). Relapse was diagnosed by routine CT-examination in only one asymptomatic patient, whereas the other relapses were diagnosed by low-cost procedures like palpation, blood tests or chest X-ray. No significant differences in age at initial diagnosis, stage or histology were seen between the two groups.

### Initial seminoma

All, except one patient (#6) presented with extra-lymphatic metastases at late relapse (Table 3). All 10 seminoma patients relapsed with viable MGCT. In eight patients pre-salvage treatment histology was available, which was separate from seminoma in three.

### Initial non-seminoma

**Figure 2:**
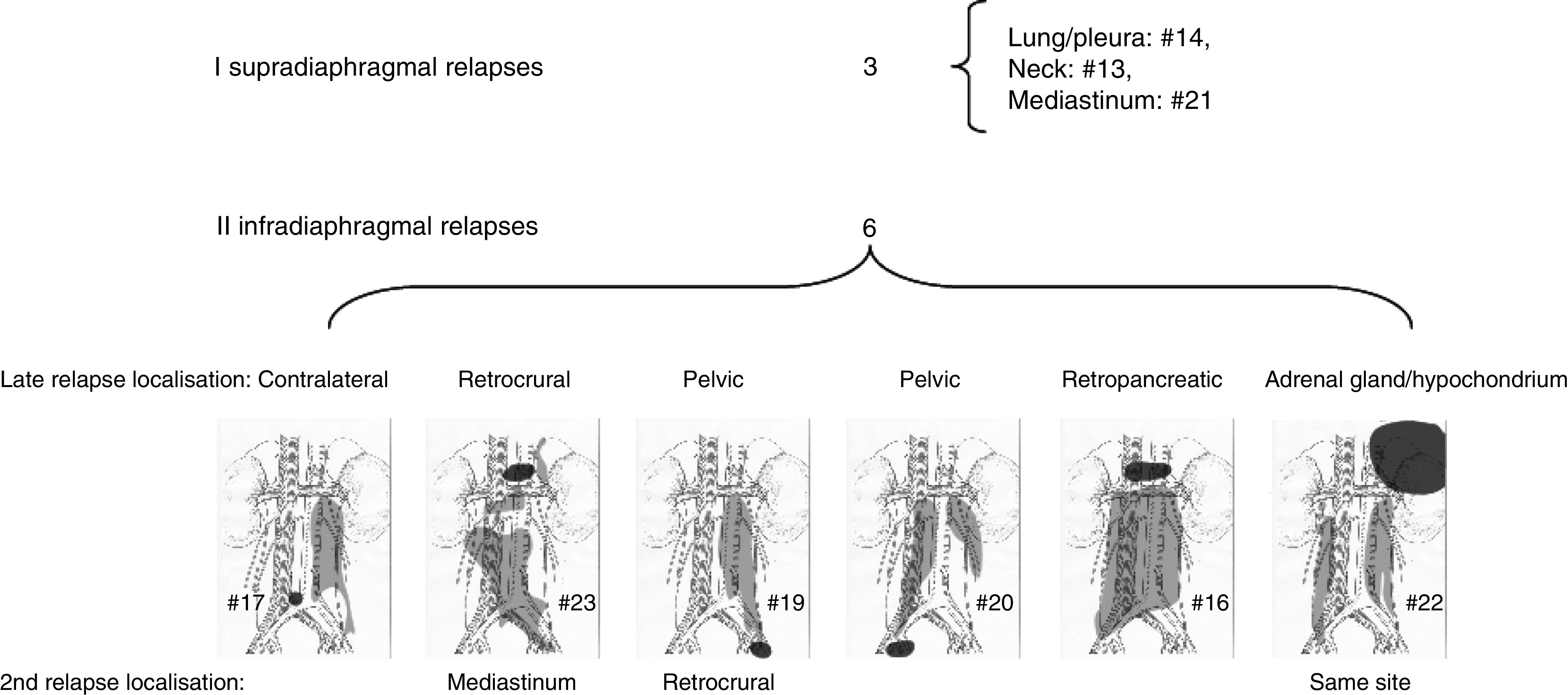
Late relapse sites of nine non-seminoma TC patients after post-chemotherapy RPLND. Sketches show operation-fields/templates in light grey and site of first relapse in dark grey.

The lungs were the only site of late relapse in three patients, all of which had had supra-diaphragmal disease at initial presentation (Table 4). In one patient (#18), the lungs and retroperitoneal lymph nodes were affected. All others had lymph node metastases only. Three of four TC patients with no primary RPLND relapsed retroperitoneally, including the surveillance patient (#11, #12, #18). Of nine TC patients with primary postchemotherapy RPLND, six presented with retroperitoneal relapses, all located close to, but outside the operation field (Figure 2).

Of nine patients with initial chemotherapy and normal tumour markers, seven relapsed with teratoma (*n*=6) or necrosis (#14) and two with viable malignant tumour. The presence of viable malignant tumour was more frequent in initial seminoma- than in initial non-seminoma patients (10 out of 10, eight out of 15, Fisher's Exact, *P*=0.02).

### Treatment

All 10 seminoma patients and seven of the eight non-seminoma patients with viable malignant tumours received salvage chemotherapy. Eight of the 10 tumour-marker-negative non-seminoma patients underwent surgery alone, whereas the remaining two (#11, #22) received subsequent chemotherapy. Chemotherapy represented the initial step of salvage treatment in four of five non-seminoma patients with elevated HCG/AFP. The fifth patient (#8) underwent surgery, despite elevated serum HCG. The rationale behind was a spontaneous drop in serum HCG before any therapeutic intervention, suggesting intermittent leakage from a cystic teratoma (van der Gaast *et al*, 1991; Ustun *et al*, 2002).

Eight patients did not undergo surgery for their late relapse. Seven out of these had initial seminoma and received a combination of chemotherapy and radiotherapy. The remaining one patient (#25), with local recurrent primary abdominal EGGCT, was the only one in our series who was solely treated by chemotherapy. There was no significant difference in survival between these eight patients and the remaining 17 who underwent surgery as part of relapse treatment (log-rank, *P*=0.24). Eleven of the operated patients had teratoma or necrosis and were rendered tumour-free by surgery alone. Of the remaining six with viable malignant tumour, three (#8, #11 and #24) could be operated radically. A radical operation was not feasible in the other three cases (#15, #16 and #22).

### Outcome and survival

Twenty-two out of 25 patients (88%) were considered tumour-free after treatment of the first late relapse. Seven patients experienced a second relapse, four of them with viable malignant tumour (#1, #10, #22 and #25).

**Figure 3:**
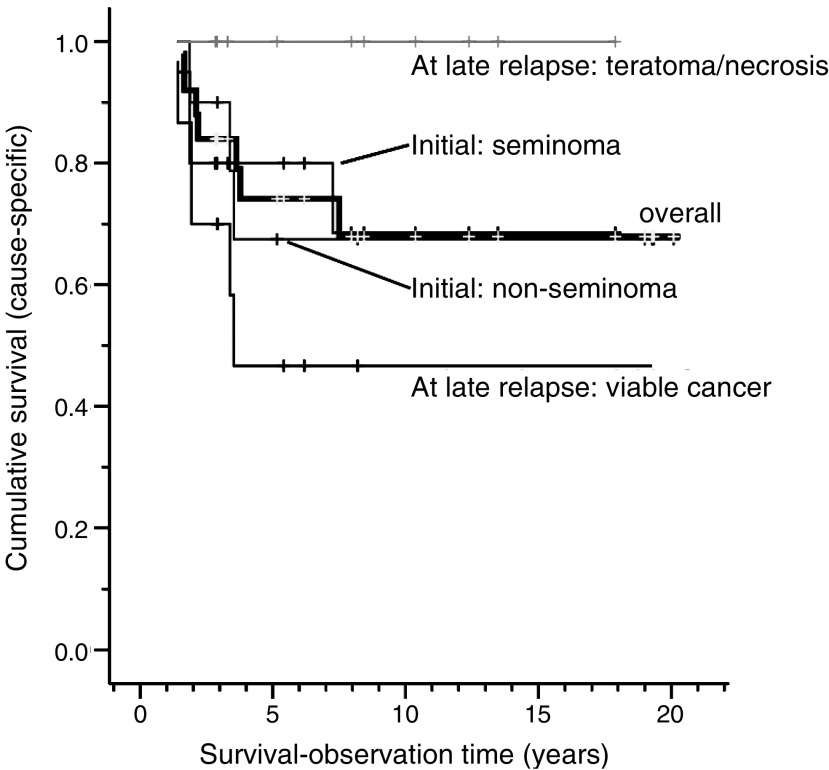
Cumulative survival according to initial and late-relapse histology.

After a median observation-time of 74 months (range: 17–241 months), 16 patients were alive without MGCT and seven patients were dead of their malignancy. Thus, the 10-year postrelapse cause-specific survival was 68 % (95% CI: 48–88%, Figure 3).

Teratoma or necrosis at late relapse (before or after salvage chemotherapy, *n*=11) conferred a 100% cause-specific survival, compared to a 50% cause-specific survival in the remaining 14 patients with viable malignant tumour (95% CI: 14–79%, log-rank test, *P*=0.009, Figure 3). The former 11 patients with favourable histology could all be operated radically, whereas all three with incomplete resection had viable malignant tumour and died within 2 years (#15, #16, #22).

Factors without statistical significance for survival were: the presence of symptoms at late relapse (*P*=0.65), initial seminoma *vs* non-seminoma (*P*=0.99), UICC-stage (*P*=0.80), IGCCCG-prognosis-group (*P*=0.67), chemotherapy-naivety at late relapse (*P*=0.83), initial presence of teratoma (*P*=0.91), and the integration of surgery into the treatment of late relapse (*P*=0.24).

## DISCUSSION

In this large population-based series of 1949 patients with MGCT, only 25 patients (1.3%) with relapses were identified. All 10 seminoma and eight of 15 non-seminoma patients relapsed with viable malignant tumour. The cause-specific postrelapse 10-year survival was 68% in all patients, 50% in patients with vital malignant tumour at late relapse and 100% in patients with teratoma or necrosis before or after salvage chemotherapy. Initial histology or the presence of symptoms was of no prognostic importance.

Our incidence rate of 1.3% is considerably lower than figures from the literature (2.9% (Baniel *et al*, 1995), 4.3% (Gerl *et al*, 1997), 5.9% (Shahidi *et al*, 2002)). These discrepancies may be due to referral and publication bias in that patients with poor prognosis, and thus, at high risk of late relapses, may preferably have been referred to experienced centers (Aass *et al*, 1991; Collette *et al*, 1999), which regularly publish their results. A referral-bias may also explain the finding of 40% initial seminoma in late relapses compared to 2–4% in the series from the Indiana group (Baniel *et al*, 1995; George *et al*, 2003), 4% in a German series (Gerl *et al*, 1997), or to 28% in a series from the Royal Marsden Hospital (Shahidi *et al*, 2002). The latter series was comprised solely of patients who had their primary treatment at the respective hospital. Referral-bias is excluded when the incidence of late relapse is calculated in population-based cohorts, as in the present series.

At our institution, postchemotherapy RPLND is performed routinely in all patients who initially present with retroperitoneal lymph node metastases, including cases of minimally residual disease (Fossa *et al*, 1989, 1992; Oldenburg *et al*, 2003). This aggressive surgical approach as part of primary treatment may to some extent explain the low risk of late relapses in our patient population. Stephenson and Sheinfeld support this view by their review on the impact of RPLND in the management of TC, which shows that relapses after primary RPLND in stage I/II non-seminomas are rather rare, cisplatin-sensitive, and extra-retroperitoneal versus more frequent and predominantly retroperitoneal, cisplatin-resistant relapses after primary chemotherapy alone (Stephenson and Sheinfeld, 2004).

Strategies to minimise the retroperitoneal operation field by limited and nerve-sparing RPLND may have increased the risk of relapses. Six of nine patients relapsed retroperitoneally after introduction of this strategy. Although we do not have a control group to compare with, it seems prudent to assume that some of these relapses may have been avoided by more extensive surgical approaches in the first place. However, a principal change from the ‘nerve sparing’ procedure would imply a major increase in side effects like retrograde ejaculation (Jacobsen *et al*, 1999).

Four of 10 initial seminoma patients relapsed as non-seminoma, one non-seminoma patient relapsed as seminoma, and three relapses comprised undifferentiated carcinoma, which may represent somatic transformation in teratomas. Thus, the initial histology is an unreliable predictor of the late relapse histology. However, the histological diagnosis of late MGCT in late relapses may be difficult, as evident in our cases #24 and #25, in whom the diagnoses were revised from adenocarcinoma to yolk-sac tumour (confer Appendix A). One of the yolk-sac tumours was of the parietal type, a probably chemotherapy-induced feature (Damjanov *et al*, 1984; Michael *et al*, 2000).

Before applying salvage chemotherapy in patients considered to have a late MGCT relapse, we strongly recommend to verify the assumed diagnosis histologically by a large, representative biopsy or, if feasible, by the completely removed lesion (Nichols, 1999). The latter approach led to cure in eight of our non-seminoma patients. AFP/HCG elevation in patients strongly suggests the presence of MGCT, and cisplatin-based chemotherapy may be given first, followed by a complete resection of residual masses, if necessary, by multiple advanced surgical procedures (Murphy *et al*, 1993). Nevertheless, a presalvage chemotherapy biopsy may prove invaluable even in patients with elevated serum tumour markers, since HCG or AFP elevation may occur unrelated to MGCT. Furthermore, one might consider omitting post-chemotherapy surgery in case of pure seminoma in the late relapse.

A survival benefit of asymptomatic patients with late relapses as reported by George *et al* (2003) could not be confirmed. Some investigators advocate regular life-long follow-up of *all* patients with MGCT (Baniel *et al*, 1995; Gerl *et al*, 1997; George *et al*, 2003). However, clinicians from the Royal Marsden Hospital consider a 5-year follow-up sufficient in TC patients except in those presenting with metastatic non-seminoma (Shahidi *et al*, 2002). A German multicentre study recommends 10-year follow-up risk of risk patients, for example, those with previous relapse, teratoma, or chemotherapy-requiring metastases (Dieckmann *et al*, 2005). We consider the latter approach as reasonable, since relapses after 10 years were very rare. Two patients from the present series (#16 and #22) might have especially benefited from such extended follow-up as their lesions probably have advanced uninhibited over years until symptoms coincided with inoperability. It seems that primary chemotherapy-requiring patients and those with EGGCT were at particularly high risk for late relapses. These should be considered for prolonged, at least 10-years, follow-up. Thereafter, annual routine check-ups may be arranged in conjunction with general practitioners with regard to late relapses, second cancers, and treatment-induced metabolic changes (Vaughn *et al*, 2002; Sagstuen *et al*, 2005; Travis *et al*, 2005).

The 10-year cause-specific survival rate of 68% for our 25 patients is promising when compared to most other reports (Baniel *et al*, 1995; Gerl *et al*, 1997; George *et al*, 2003). Except for the small number of patients, a possible explanation for the favourable survival-rate may be that our series is comprised of *all* patients with late relapses, both those recurring after initially advanced stages and those with stage I, and includes both initial seminoma and non-seminoma patients.

In summary, the low incidence-rate of 1.3% is probably best explained by the population-based approach of *all* patients with MGCT, treated at one experienced oncological unit, and by the aggressive treatment of the initial MGCT. All 10 seminoma patients, but only eight of 15 non-seminoma patients, relapsed with viable malignant tumour. The 10-year survival rate in all patients was 68%, 100% in those with teratoma/necrosis, and 50% in those with vital malignant tumours.

## Figures and Tables

**Table 1:** Treatment strategies

	**Period**	**Stadium I and II (small volume)^a^**	**Stadium II (high-volume)^b^, III and IV**
Seminoma	1971–1997	Infradiaphragmal radiotherapy	*Chemotherapy*
			Alkylating agents<1980
			Cisplatin-based>1980
			
			*Followed by*
			Radiotherapy or surgery<1988
			
Non-seminoma	1980–1989	Primary RPLND (retroperitoneal lymph node dissection) (unilateral template)+Cisplatin-based chemotherapy in case of metastases	Induction chemotherapy, followed by surgical removal of residual masses: bilateral template RPLND<1985, unilateral RPLND, if possible>1985
		(borders for unilateral template: aortic midline, ipsilateral ureter, renal vein, bifurcation of the ipsilateral iliac artery)	(borders for bilateral RPLND: ureters, the renal veins and the ipsilateral iliac bifurcation)
			
	1990–1997	Stage I: low risk^c^: wait and see, high risk^d^: adjuvant chemotherapy	Stage II (all)–IV chemotherapy, followed by surgical removal of all residual masses, even in case of ‘normal’ abdominal CT, by nerve-sparing RPLND, if possible
EGGCT	1980–1997	Cisplatin-based chemotherapy, followed by surgical removal of residual masses, exceptionally radiotherapy only in a single small seminomatous mass	

a <3 cm.

b ⩾3 cm.

c No vascular invasion, orchiectomy specimen.

d Vascular invasion, orchiectomy specimen.

**Table 2:** Demographics in 25 patients with late relapses of MGCT, by mode of detection

	**All**	**Symptoms**	**Routine**
*n* (%)	25 (100)	14 (56)	11 (44)
Age^a^ (years)	30 (20–68)	27 (20–48)	36 (23–68)
			
*Primary histology*			
Seminoma	10	6	4
Non-seminoma	15	8	7
			
*UICC stage* b			
I	3	2	1
IS	2	1	1
II	6	4	2
III	10	6	4
Extragonadal	4	1	3
			
*IGCCCG*^c^ *category stage*			
Good	11	7	3
Intermediate	5	2	4
Poor	3	1	2
			
*Chemotherapy in primary treatment*			
Seminoma	3	1	2
Nonseminoma	14	7	7
			
Months to relapse^a^	55 (32–224)	88 (40–224)	41 (32–110)
			
*Sites of relapse*			
Retroperitoneal	9	6	3
Mediastinum	6	3	3
Lung/pleura	5	2	3
Neck/supraclavicular	3	1	2
Retrocrural	1	1	
Pelvis	3	2	1
			
Number of relapse sites	27^d^	15^d^	12^d^
			
*Symptoms leading to unscheduled visit*			
Tiredness		4	
Pain (back/abdominal)		6 (3/3)	
Dyspnoea		2	
Peripheral oedema		1	
Dysphagia		1	
			
*Findings at routine follow-up leading to diagnosis*			
Radiology (chest X-ray/CT-thorax)			4 (3/1)
Elevated markers (AFP/HCG)			4 (2/2)
Palpable masses (supraclv./pelvis)			3 (2/1)
Diameter (mm)^a^	35 (10–135)	43 (11–135)	20 (10–46)
			
*Status*			
NED^e^	16	10	6
DOD^e^	7	3	4
DID^e^	2	1	1

a Median (range).

b International Union against cancer (Sobin and Wittekind, 2002).

c International Germ Cell Consensus Classification Group (1997).

d Three patients experienced the late relapse at two sites, as shown in Tables 2 and 3.

e Status: NED alive, no evidence of disease; DOD, dead of disease (MGCT); DID, dead of intercurrent disease, tumour-free.

**Table 3:**
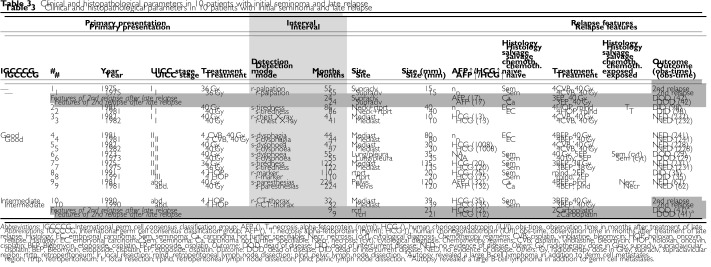
Clinical and histopathological parameters in 10 patients with initial seminoma and late relapse

**Table 4:**
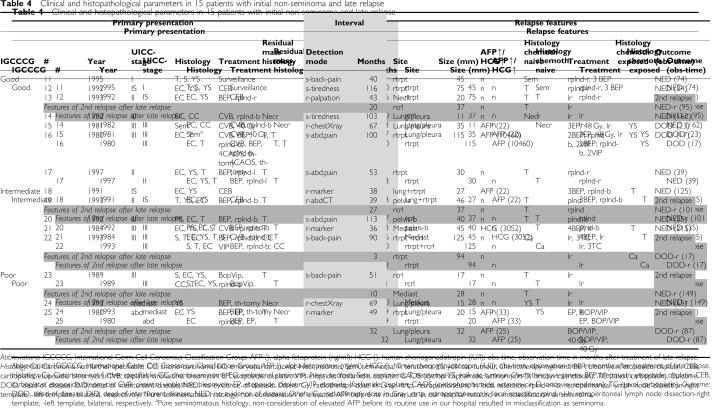
Clinical and histopathological parameters in 15 patients with initial non-seminoma and late relapse

## Appendix A

*Patient # 4* suffered from dysphagia and was, based on primary histology, considered to have a primary oesophageal cancer, until expert review of the histological specimen at the NRH led to the diagnosis of a late relapsing TC.

*Patient # 8* had a asymptomatic retroperitoneal relapse, which was detected by elevated serum HCG during a research-based long-term survey 10 years after primary treatment (Fossa *et al*, 2003).

In *patient # 14*, a routine chest X-ray performed as part of routine check-up, revealed a new small mass in the left lower lobe. The histological examination of the thoracotomy specimen revealed a completely necrotic MGCT metastasis.

*Patient # 24*, with an initial mediastinal EGGCT, relapsed after 5¾ years with a single 15 mm mass in the left lung and normal serum markers. The initial histological diagnosis of the removed nodule was adenocarcinoma, with a weak AFP staining. However, review of the histology performed during the present study revealed that the mass was a yolk sac tumour.

In *patient # 25*, a mediastinal tumour and a cyst in the neck had been removed in 1977, revealing teratoma in both cases. In 1980 he developed malignant spinal cord compression due to a retroperitoneal mass, primarily categorised as ‘unclassifiable carcinoma’. After referral to the NRH, the case history, an elevated serum AFP level and the review of the histology led to the diagnosis of a recurrent EGGCT.
